# Assessment of Addictive Behavior in Rats with Partial Knockout of the Dopamine Transporter Gene

**DOI:** 10.3390/ijms27125604

**Published:** 2026-06-21

**Authors:** Andrey A. Lebedev, Petr D. Shabanov, Elena E. Lyakso, Olga V. Frolova, Egor A. Kleshnev, Aleksandr S. Nikolaev, Vadim V. Sizov, Maria A. Netesa, Ivan A. Balaganskii, Sarng S. Pyurveev

**Affiliations:** 1 Department of Higher Nervous Activity and Psychophysiology, St. Petersburg State University, 29 16th Line of Vasilyevsky Island, St. Petersburg 199178, Russialyakso@gmail.com (E.E.L.);; 2 Department of Pharmacology, Institute of Experimental Medicine, 12 Akademika Pavlova St., St. Petersburg 197022, Russiabalaganskiiivan@mail.ru (I.A.B.)

**Keywords:** addictive behavior, dopamine transporter, DAT-HET, extracellular dopamine, impulsivity, compulsivity, self-stimulation, VR ratio

## Abstract

Animals with knockout of the dopamine transporter gene (DAT-KO) display hyperdopaminergic phenotypes, including attention-deficit/hyperactivity-like behaviors. A previous behavioral analysis of heterozygous rats with partial knockout (DAT-HET) suggested increased susceptibility to addictive behaviors. The aim of this study was to investigate elements of addictive behaviors and the mechanisms underlying dopamine release in DAT-HET rats. Offspring derived from DAT-knockout breeding underwent genotyping and behavioral assessment using the marble burying test, a manipulative behavior test using nesting material, and a modified version of the Iowa Gambling Task. Feeding behavior was studied using a binge-eating model. Reinforcing properties were investigated using intracranial self-stimulation under fixed-ratio (FR) and variable-ratio (VR) schedules. Dopamine (DA) release and clearance dynamics were assessed using fast-scan cyclic voltammetry (FSCV). DAT-HET rats exhibited moderate hyperactivity, increased impulsive choice, and compulsive responses. Male DAT-HET rats also showed increased compulsive overeating compared with wild-type (WT) rats of both sexes and female DAT-HET rats. In addition, DAT-HET rats demonstrated a preference for VR self-stimulation, which resembles risk- and thrill-seeking behavior in humans. In DAT-KO rats, impaired DA clearance resulted from complete loss of dopamine transporter function. In DAT-HET rats, increased DA release amplitude was observed, and dopamine persisted longer in the extracellular space than in WT rats. These findings underscore the importance of the DAT-HET model for studying impulsivity, compulsivity, and factors underlying the predisposition to addictive behavior.

## 1. Introduction

The dopaminergic system plays a key role in the regulation of behavior by integrating motivational, emotional, and cognitive processes mediated by the prefrontal cortex, striatum, and mesolimbic system [1,2]. Disruption of dopaminergic transmission results in functional reorganization of systems involved in adaptive behavior [3,4].

The dopamine transporter (*DAT*) controls extracellular dopamine levels. Inactivation of *DAT* leads to marked hyperdopaminergia, loss of autoregulatory control, and altered sensitivity of D1 and D2 receptor systems [5,6]. DAT-KO animals exhibit behavioral features characteristic of attention-deficit/hyperactivity disorder (ADHD), including hyperactivity, reduced attention, and cognitive impairments [7].

Dysfunction of the dopaminergic system increases susceptibility to addictive behaviors [8]. DAT-KO animals exhibit increased exploratory activity and sensitivity to rewarding stimuli [9].

The concept of behavioral addiction (non-substance-related addiction) in the current DSM-5 and ICD-11 classifications has evolved substantially, reflecting the recognition that certain activities can induce addiction in a manner analogous to psychoactive substances. Experimental models of addictive behavior, particularly those assessing impulsivity and compulsivity, allow assessment of behavioral regulation disorders [10]. Addictive eating disorders, such as binge eating, are also of great interest [11].

Impulsivity and compulsivity are behavioral traits that directly influence decision-making. Impulsivity can be described as a tendency to engage in poorly controlled and risky actions without adequately considering possible negative consequences [12]. Impulsivity has two key components: impulsive motor responses (motor impulsivity) and impulsive choice [13]. Impulsive actions are characterized by premature responses resulting from an inability to inhibit them. Impulsive choice manifests in decision-making that prioritizes immediate rewards over more advantageous delayed rewards [14]. Thus, impulsive choice reflects an irrational evaluation of possible alternatives.

Compulsivity is viewed as a tendency toward repetitive, stereotyped behavioral acts aimed at alleviating negative affective states, anxiety, and stress [15]. Such behavior is often uncontrollable and may lead to negative consequences. Elements of compulsive behavior are widespread and are observed in obsessive–compulsive disorder, addictive disorders, and eating disorders [16]. Like impulsivity, compulsivity is characterized by impaired decision-making and a deficit in inhibitory cognitive control associated with reduced function of the prefrontal cortex, anterior cingulate cortex, and ventral striatum [15].

Binge eating disorder (BED) is the most common eating disorder [17]. It is characterized by binge eating (at least one episode per week for 3 months), accompanied by emotional distress and occurring without compensatory eating behaviors. Lifetime prevalence of BED is approximately 1.4%. BED is more common in women than in men [17]. Up to 30% of patients with obesity meet the criteria for BED [18]. BED has a genetic predisposition and a polygenic inheritance pattern. The heritability of BED is estimated at approximately 45%; the remaining factors can be attributed to environmental dispersion [19].

Elements of addictive behavior were also investigated through stimulation of positive reinforcement regions in the brain [20]. The animals were first trained to perform self-stimulation using a two-pedal method with a choice between variable ratio (VR) and fixed ratio (FR) reinforcement schedules [20]. The VR schedule has been shown to most closely approximate the thrill and risk associated with gambling in humans [21].

Heterozygous animals with partial dopamine transporter deficiency (DAT-HET) are of particular interest as a model of moderate and chronic hyperdopaminergia. Unlike DAT-KO, in which an extremely sharp increase in extracellular dopamine is observed, DAT-HET is characterized by a milder but stable decrease in dopamine reuptake efficiency. This is accompanied by increased motor activity, changes in impulsive and compulsive behavior, as well as impaired behavioral flexibility [5,22,23].

Research indicates that it is precisely a partial *DAT* deficiency that can to a certain extent model subclinical and premorbid states in which the general organization of behavior is preserved, but the balance between the motivational and cognitive components of action regulation is disrupted [7]. This makes DAT-HET animals a promising model for analyzing early and compensated forms of reward system dysregulation. Considering the complexity of the human mental sphere analysis of this model may help to a certain extent clarify the mechanisms underlying the development of impulsive and compulsive elements of behavior and identify neurobiological markers of vulnerability to addictive behavior. However, no systematic studies of elements of addictive behavior in rats with partial *DAT* deficiency have been reported to date. The present study aims to identify stable genotype-dependent differences in the behavioral phenotypes of DAT-HET and wild-type (WT) animals. To study the mechanisms of addictive states, phasic increases in extracellular dopamine levels in the nucleus accumbens (NAc) in response to stimulation of the ventral tegmental area (VTA) were analyzed [8].

Research objective. To study manifestations of addictive behaviors and the underlying mechanisms of dopamine release in DAT-HET rats.

## 2. Results

### 2.1. Assessment of Compulsive and Stereotypical Behavior in Rats with Incomplete Dopamine Transporter (DAT) Knockout

To assess the severity of compulsive behavior in rats, a marble-burying test was conducted. Significant genotype-dependent differences were observed between WT and DAT-HET rats. According to the results of one-way analysis of variance, the number of buried marbles depended significantly on the rat’s genotype (ANOVA: F (2, 24) = 5.862; *p* = 0.0085), indicating that the degree of dopamine transporter dysfunction influences the severity of compulsive behavior.

**Figure 1:**
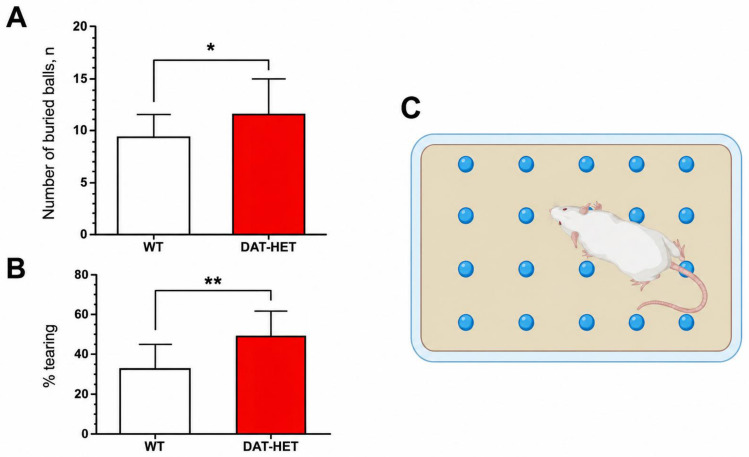
Compulsive-like behavior in wild-type (WT) and dopamine transporter heterozygous knockout (DAT-HET) rats. (**A**) Number of buried marbles in the marble burying test. (**B**) Percentage of nest material shredded in the nestlet shredding test. (**C**) Schematic representation of the marble burying apparatus. Data are presented as mean ± standard deviation (SD). * *p* < 0.05, ** *p* < 0.01 compared with WT rats.

Wild-type (WT) rats buried an average of 9.90 ± 1.20 marbles, corresponding to a low level of stereotypic-compulsive activity. DAT-HET heterozygotes showed a significantly higher index, reaching 12.20 ± 2.15 marbles (Figure 1A).

A subsequent Tukey post hoc analysis confirmed the presence of significant differences between WT and DAT-HET: *p* = 0.0409.

Statistically significant differences among the animal groups were identified in the nest material shredding test. One-way analysis of variance showed that the degree of nest material shredding was significantly dependent on genotype (Figure 1B). (ANOVA: F(1, 36) = 7.453; *p* = 0.0030), indicating that the functional state of the dopamine transporter influences the severity of compulsive activity.

Wild-type (WT) rats exhibited the lowest level of compulsive behavior, shredding an average of 34.90 ± 8.23% of the material. In *DAT* knockout heterozygotes (DAT-HET), the values were significantly higher, reaching 51.80 ± 7.22%.

A post hoc Tukey analysis revealed a significant difference between WT and DAT-HET (*p* = 0.0021). A compact representation of group differences showed that DAT-HET form a distinct phenotypic cluster differing from WT.

### 2.2. Assessment of Impulsive Behavior in Rats with Partial Dopamine Transporter (DAT) Knockout

Analysis of the total number of runs revealed marked genotype-dependent differences in the animals’ locomotor activity to achieve food reinforcement in a maze. One-way analysis of variance showed a significant effect of genotype on the level of locomotor activity to achieve food reinforcement in a maze (F(1, 36) = 81.18; *p* < 0.0001).

**Figure 2:**
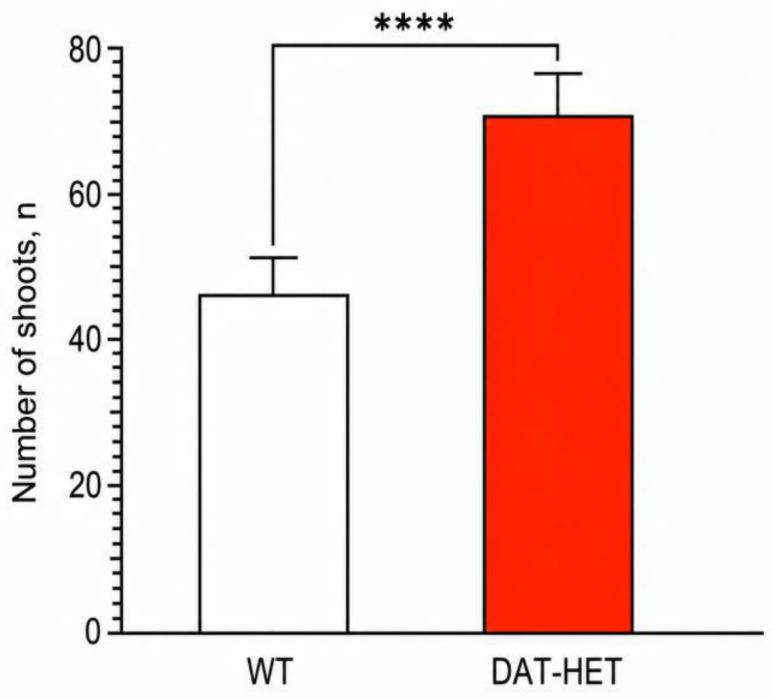
Motor activity to achieve food reinforcement of wild-type (WT) and dopamine transporter heterozygous knockout (DAT-HET) rats during the Iowa Gambling Task. The total number of runs performed during the testing session is shown. Data are presented as mean ± standard error of the mean (SEM). **** *p* < 0.0001 compared with WT rats.

Wild-type rats exhibited a minimum number of runs to achieve food reinforcement in a maze of 46.10 ± 3.96. In heterozygotes for the dopamine transporter knockout (DAT-HET), the value was significantly higher at 69.70 ± 6.38 (Figure 2).

A post hoc Tukey analysis confirmed a significant increase in activity in the heterozygotes: WT vs. HET: *p* < 0.0001.

### 2.3. Genotype-Dependent Organization of Choice Strategies in the Iowa Gambling Task

**Figure 3:**
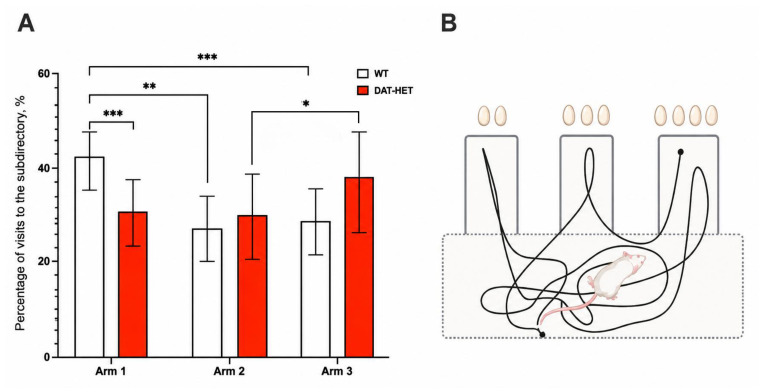
Choice behavior in the Iowa Gambling Task in wild-type (WT) and dopamine transporter heterozygous knockout (DAT-HET) rats. (**A**) Percentage of visits to each arm of the maze. Arm 1 was associated with a low reward magnitude and a high probability of reinforcement, whereas Arm 3 was associated with a high reward magnitude and a low probability of reinforcement. Data are presented as mean ± standard error of the mean (SEM). Statistical analysis was performed using a two-way repeated-measures ANOVA, with genotype as the between-subject factor and arm as the within-subject repeated-measures factor, followed by Tukey’s multiple comparisons test. WT rats exhibited a preference for Arm 1 compared with Arms 2 and 3, whereas DAT-HET rats differed between Arm 2 and Arm 3. * *p* < 0.05, ** *p* < 0.01, *** *p* < 0.001. (**B**) Schematic representation of the Iowa Gambling Task apparatus illustrating the trajectory of a representative animal, and symbols indicate the reward locations in the maze arms.

A two-way repeated-measures ANOVA, with genotype as the between-subject factor and arm as the within-subject repeated-measures factor, revealed a significant genotype × arm interaction (F(2, 72) = 7.35, *p* < 0.0001), as well as a significant main effect of arm (F(2, 72) = 6.71, *p* = 0.0021). Post hoc Tukey’s multiple comparisons test showed that WT rats exhibited a marked preference for Arm 1 compared with Arms 2 and 3 (*p* < 0.0001), whereas DAT-HET rats differed between Arm 2 and Arm 3 (*p* = 0.0495) (Figure 3A,B).

### 2.4. Assessment of Binge Eating

**Figure 4:**
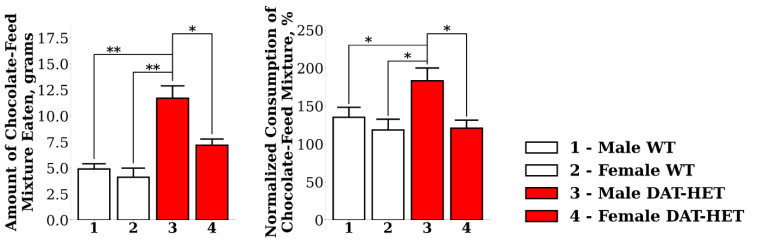
Total (**left**) and normalized (**right**) consumption of the chocolate-feed mixture by wild-type (WT) rats and dopamine transporter knockout heterozygotes (DAT-HET). Values are presented as mean ± standard deviation. *—*p* < 0.01, **—*p* < 0.001.

The experiments revealed that WT males consumed more of the chocolate mixture on average (4.9 ± 0.5 g, *p* < 0.001) compared to WT females (4.1 ± 0.9 g, *p* < 0.001). Meanwhile, DAT-HET males consumed significantly more of the chocolate mixture on average (11.7 ± 1.2 g) compared to both WT males (4.9 ± 0.5 g, *p* < 0.001) and WT females (4.1 ± 0.9 g, *p* < 0.001), as well as DAT-HET females (7.2 ± 0.6 g, *p* < 0.01). The normalized increase in chocolate treat consumption was also calculated. For WT males, this indicator was 135.3 ± 13.1%, while for WT females, it was 118.7 ± 14.0%. For DAT-HET males, this parameter was significantly higher (183.3 ± 16.9%), relative to both WT males (*p* = 0.048) and DAT-HET females (121.0 ± 10.7, *p* = 0.021) (Figure 4).

### 2.5. Investigation of the Addictive Component of Self-Stimulation Behavior

**Figure 5:**
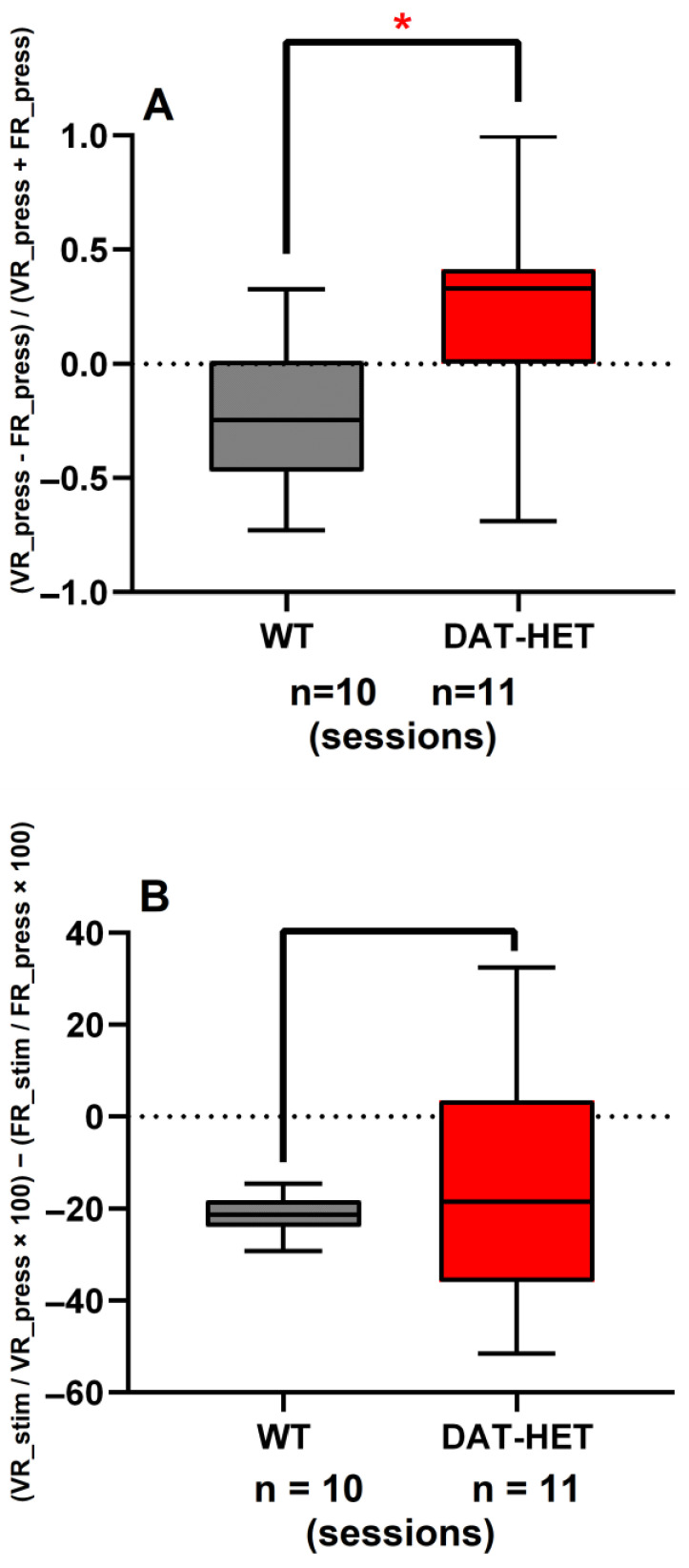
Exploratory session-level analysis of response distribution between FR- and VR-associated levers in the intracranial self-stimulation task. Each point in the graph corresponds to an individual session. (**A**) Response distribution was assessed using the press preference index, calculated according to Formula 1: PPI = (VR_press − FR_press)/(VR_press + FR_press). Positive PPI values indicate a predominance of responses on the VR-associated lever, whereas negative values indicate a predominance of responses on the FR-associated lever. In the session-level analysis, PPI values were higher in the DAT-HET group compared with WT: WT, *n* = 10 sessions, median = −0.245, mean = −0.218; DAT-HET, *n* = 11 sessions, median = 0.329, mean = 0.225. The difference reached statistical significance according to the two-tailed Mann–Whitney U test: *p* = 0.0159; Cliff’s delta = 0.618. (**B**) As a control for actual reinforcement density, actΔ(VR − FR) was calculated according to Formula 2: actΔ(VR − FR) = (VR_stim/VR_press × 100) − (FR_stim/FR_press × 100). This measure reflects the difference between the VR and FR schedules in the number of stimulations per 100 responses. actΔ values did not differ significantly between groups: median WT = −21.27; median DAT-HET = −18.44; two-tailed Mann–Whitney U test, *p* = 0.426; Cliff’s delta = 0.218. * *p* < 0.05 for the WT vs. DAT-HET comparison.

Figure 5A shows the primary behavioral measure, PPI, calculated according to Formula 1. Figure 5B shows the control measure, actΔ(VR − FR), calculated according to Formula 2 and reflecting the actual reinforcement density per 100 responses.

In an exploratory session-level analysis, PPI values in the DAT-HET group were higher than those observed in WT sessions. In WT animals, the distribution of responses was shifted toward the FR-associated lever: the median PPI was −0.245, and the mean value was −0.218, based on 10 sessions from 5 animals. In contrast, DAT-HET sessions showed a predominance of VR-associated responses: the median PPI was 0.329, and the mean value was 0.225, based on 11 sessions.

In the session-level analysis, the difference between WT and DAT-HET reached statistical significance according to the two-tailed Mann–Whitney U test: *p* = 0.0159. Cliff’s delta was 0.618, consistent with a predominance of higher PPI values in DAT-HET sessions.

Median actΔ values were similar between groups: −21.27 in WT sessions and −18.44 in DAT-HET sessions. No statistically significant between-group difference was detected: two-tailed Mann–Whitney U test, *p* = 0.426, Cliff’s delta = 0.218.

Additionally, the total number of responses during the 5 min period after the first response was analyzed. The median total number of responses was 234.5 in WT sessions and 186.0 in DAT-HET sessions. The between-group difference did not reach statistical significance: two-tailed Mann–Whitney U test, *p* = 0.377.

### 2.6. Assessment of Extracellular Dopamine in the Nucleus Accumbens

**Figure 6:**
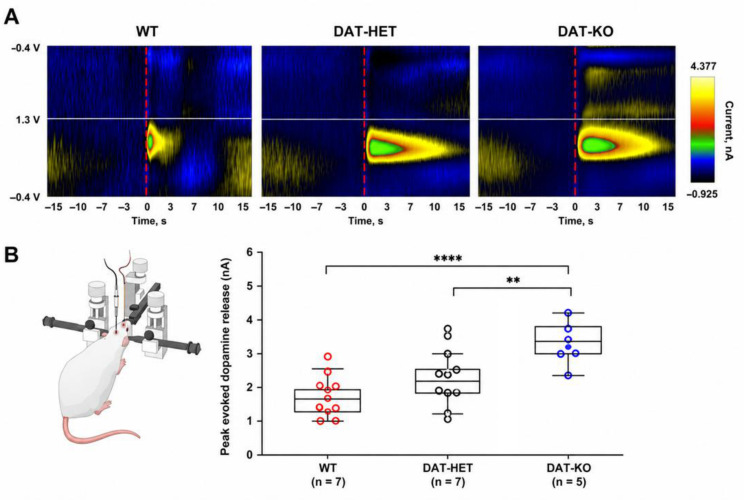
Extracellular dopamine dynamics in the nucleus accumbens (NAc) assessed by fast-scan cyclic voltammetry (FSCV) following electrical stimulation of the ventral tegmental area (VTA) in rats with different dopamine transporter genotypes (WT, DAT-HET, and DAT-KO). (**A**) Representative FSCV pseudocolor plots illustrating genotype-dependent differences in dopamine release and clearance. The red dashed line indicates the onset of VTA stimulation. Color intensity represents changes in oxidation current associated with extracellular dopamine concentration over time and applied potential for each genotype, reflecting genotype-dependent differences in dopamine release and clearance. (**B**) Schematic representation of the FSCV recording configuration (**left**) and quantitative analysis of peak evoked dopamine release in the NAc (**right**). Individual data points are shown together with median and interquartile range. Statistical analysis was performed using the Kruskal–Wallis test followed by Dunn’s multiple comparisons test. Significant differences were observed between DAT-KO and WT rats (** *p* < 0.01) and between DAT-KO and DAT-HET rats (** *p* < 0.01), whereas no significant differences were detected between WT and DAT-HET animals. WT, *n* = 7; DAT-HET, *n* = 7; DAT-KO, *n* = 5. **** *p* < 0.0001.

In rats under free-behavior conditions, extracellular dopamine levels were measured using FSCV in the nucleus accumbens (NAc) in response to stimulation of the VTA. Extracellular dopamine release was investigated in WT, DAT-KO, and DAT-HET rats. The presented pseudo-colored graphs demonstrate characteristic differences in dopaminergic transmission between the groups (Figure 6).

In the experiment measuring extracellular dopamine levels in the NAc in response to stimulation of the VTA, as determined by electrochemical potential readings, the Kruskal–Wallis test revealed significant intergroup differences in the effects of the studied dopamine levels in DAT-KO rats (H(3) = 15.44; *p* < 0.0001). Subsequent Dunn’s multiple comparison test revealed differences between the medians of dopamine release in DAT-KO animals relative to the WT animals with baseline dopamine release (*n* = 7, *p* < 0.05) and relative to the DAT-KO (*n* = 5, *p* < 0.00001) (Figure 6).

In WT rats, electrical stimulation elicited a moderate dopamine release with a maximum signal amplitude of approximately 1–1.5 nA. On voltammograms, this response manifested as a local increase in current at dopamine oxidation potentials (around 0.6 V). Dopamine clearance was rapid, with signal intensity returning to baseline within a few seconds after stimulation. This profile corresponds to normal dopamine transporter function, ensuring efficient dopamine reuptake.

In animals with a *DAT* gene knockout, a marked increase in the amplitude of dopamine release was observed, with signals exceeding 2 nA and occupying a significantly larger area on the pseudo-color diagram. Following stimulation, extracellular dopamine persisted for an unusually long period: the yellow-orange region, corresponding to elevated dopamine levels, remained for tens of seconds. The absence of a rapid decline in the signal indicates significantly impaired clearance, resulting from the complete loss of dopamine transporter function.

In DAT-HET animals, a marked increase in the amplitude of dopamine release was observed, with signals exceeding 2 nA and occupying a large area on the pseudo-color diagram. Following stimulation, extracellular dopamine persisted for a certain period: the yellow-orange region, corresponding to elevated dopamine levels, remained for tens of seconds. The absence of a signal decline indicates impaired clearance resulting from partial loss of dopamine transporter function.

## 3. Discussion

This study demonstrates that partial impairment of dopamine transporter function in DAT-HET leads to a reorganization of behavioral strategies encompassing motor, impulsive, and compulsive responses. Notably, DAT-HET rats exhibited increased compulsive overeating. The differences observed between the DAT-HET and WT phenotypes are consistent with the literature. A partial *DAT* deficiency appears to result in a distinct behavioral profile characterized by moderate hyperdopaminergia combined with relative stability of behavioral strategies and lower reactivity variability [24].

In the present study, DAT-HET rats demonstrated high motor activity to achieve food reinforcement in a maze, pronounced impulsivity, and compulsivity, while showing the lowest variability among the experimental groups. This behavioral profile corresponds to a state of sustained, relatively constant elevation of baseline dopamine levels, leading to chronic hyperstimulation of D1 and D2 receptors, desensitization of receptor autoregulation, and suppression of the directionality of phasic dopamine signals necessary for encoding reward prediction errors and adequately assessing the probabilistic characteristics of reinforcement [25]. It is precisely this state, as shown in recent studies, that leads to behavioral rigidity, reduced behavioral flexibility, and the formation of persistent stereotypes [22,26].

In this context, DAT-KO can be viewed as a model of extreme manifestations of hy-perdopaminergia, accompanied by pronounced motor and cognitive impairments, whereas DAT-HET represents a more subtle model of reward system dysregulation, potentially possessing greater translational significance for studying complex forms of impulsive and compulsive behavior [27]. This interpretation is supported by the present FSCV findings demonstrating profound impairment of dopamine clearance in DAT-KO rats. In the absence of the dopamine transporter, clearance is virtually absent, the extracellular concentration of the neurotransmitter increases many-fold, and the time required for its removal from synapses increases tenfold, which is consistent with data from other electrochemical studies [28]. Such an extreme hyperdopaminergic state leads not only to receptor desensitization but also to their internal reorganization, a decrease in tyrosine hydroxylase activity, disruption of calcium/calmodulin-dependent protein kinase II phosphorylation and BDNF/TrkB signaling, as well as to dysregulation of the interaction between dopaminergic and glutamatergic transmission in the striatum [27,29,30,31].

In contrast to DAT-HET, DAT-KO mice exhibit enhanced processes of structural reorganization of dendritic spines and disrupted distribution of PSD proteins, as confirmed by morphological studies [32], which may lead to the reorganization of striatal circuits under chronic elevation of dopamine levels. This leads to disorganized hyperdopaminergia, in which behavioral programs become unstable, variable, and poorly regulated [7]. DAT-HET mice reach the threshold of dysfunction due to moderate but chronic hyperdopaminergia, which shifts the functioning of the reinforcement evaluation system: positive stimuli are underestimated, and behavior becomes faster and less calibrated. Our previous studies have shown that complete *DAT* knockout caused disorganization of behavioral regulatory mechanisms with high variability in behavioral responses [7], which may be based on a disruption of the phasic dynamics of dopamine release and changes in the receptor and molecular architecture of the striatum [29,30].

The observed increase in compulsive behaviors and manipulative behaviors involving nesting material, such as marble burying, may indicate that behavioral rigidity arises from a marked imbalance in motivational mechanisms. Under conditions of impaired regulation of the striatal D1 and D2 pathways, automated motor patterns form that are difficult to correct flexibly [33,34]. In DAT-HET, functional enhancement of D1 transmission predominates, which increases the variability of compulsive behavior [35]. In this regard, our data expand upon and underscore the value of the DAT-deficient genetic model in DAT-HET for studying the mechanisms of impulsive and compulsive behavior under conditions of probabilistic reinforcement and environmental uncertainty.

This study demonstrates the effects of compulsive overeating in DAT-HET rats. DAT-HET males consumed twice as much chocolate mixture compared to WT males. This may be explained by increased dopamine reuptake time and chronically elevated levels of extracellular dopamine. Excess dopamine may increase the “motivational significance” of food (wanting) of high-calorie food, which is associated with changes in sensitivity to reinforcing stimuli [36]. In our experiments, animals tended to continue searching for and consuming food even when accompanied by mild stress stimuli, which is a sign of compulsive behavior [37].

Differences in the behavior of male and female DAT-HET animals are apparently due to the protective effect of estrogens. They are capable of modulating dopamine transporter function and dopamine receptor activity [38]. An increase in compulsive overeating has been demonstrated in ovariectomized rats [39]. The literature provides conflicting data on gender differences in compulsive overeating. In rats without baseline alterations in the dopamine system (WT), no gender differences in compulsive overeating were observed [40]. Other studies have noted an increase in compulsive overeating in female rodents; the frequency of overeating during intermittent access was higher in females than in males [41]. Although most genetic risk factors are common to both sexes, there may be clear gender-specific genetic effects influencing susceptibility to compulsive overeating [40]. In our studies, these appear to be driven by alterations in the dopaminergic control of frontocorticostriatal circuits and may be to a certain extent associated with symptoms of attention-deficit/hyperactivity disorder (ADHD) [42]. Overeating is common among adolescents with ADHD, and one of the factors contributing to increased eating activity is likely impulsivity. Higher rates of overeating in individuals with ADHD may be explained by a baseline increase in compulsivity and impulsive behavior, impaired cognitive control, and general psychopathological factors [43].

This study compared self-stimulation response parameters under VR and FR schedules in WT and DAT-HET animals. These data suggest that, at the level of session-level observations, DAT-HET animals more frequently responded on the VR-associated lever compared with WT animals. Thus, higher PPI values in DAT-HET sessions were not accompanied by a significant increase in the actual stimulation density on the VR-associated lever. Therefore, the observed predominance of VR-associated responses cannot be explained by a higher total number of responses or by an overall increase in response rate.

Taken together, this exploratory session-level analysis showed that DAT-HET sessions were characterized by higher PPI values, reflecting a relative predominance of VR-associated responses. This effect was not accompanied by significant differences in actual reinforcement density or in the total number of responses. Therefore, the observed re-sponse distribution is unlikely to be reducible to greater availability of stimulation under the VR schedule or to a general increase in response output.

It was shown that in DAT-HET animals, there was a shift in actual reinforcement (the number of actual VTA stimulations) toward the VR schedule. When using the variable ratio (VR) schedule, the animal could not determine which press would be followed by reinforcing electrical current. This schedule most closely approximates the thrill and risk of gambling [21]. The obtained data are mostly consistent with the literature. When using a progressive ratio of self-stimulation, in which the animal was progressively required to increase the number of responses per reinforcement, DAT-KO mice showed a significantly higher maximum number of nose pokes compared to WT mice [44]. In another study using the FR1 schedule, DAT-KO mice exhibited an increase in self-stimulation frequency at lower current intensities and a delayed extinction of the response, indicating an important role for dopamine in the control of reward-related behavior [45]. No systematic studies of the characteristics of the internal reinforcement system in DAT-HET animals have been found in the literature. In our previous work, we compared the phasic dopamine release curve with a fluctuating emotional process by analyzing the dynamics of dopamine levels during self-stimulation. It was suggested that this reflects reward-based assessment processes in the regulation of approach and avoidance behavior [46], which was confirmed in the work [47] on the convergence of positive emotional stimuli of a dopamine nature and negative stimuli in neural populations of the extended amygdala, the brain substrate of addiction [48,49].

The increased reinforcing properties of electrical brain stimulation under the VR schedule compared to the FR schedule indicate a state of basal hyperdopaminergia, which influences the manifestation of addictive behavioral elements in DAT-HET animals. Since dopamine is not cleared from the synapse at the usual rate, each electrical impulse can trigger a longer lasting and stronger dopamine signal, which is subjectively perceived by the animals as more “pleasant” or meaningful. At the same time in DAT-KO animals, due to extremely high levels of extracellular dopamine, the reward system may be in a state of “constant saturation” or receptor desensitization. Studies have shown reduced sensitivity to reinforcing stimuli in DAT-KO animals [28]. In contrast, DAT-HET animals represent a “purer” model of heightened motivation. In DAT-HET, the dopamine system operates in an enhanced mode, which manifests as a clear amplification of the response to self-stimulation under the VR schedule, consistent with our data on increased impulsivity and compulsivity in their behavior.

Thus, dysfunction of the dopamine transporter leads to a marked disruption in behavioral regulation, characterized by a predominance of addictive behaviors [50]. DAT-HET subjects demonstrated a consistent and structured hyperdopaminergic pattern: increased motor activity to achieve food reinforcement in a maze, pronounced impulsivity, and compulsivity, increased compulsive overeating, and a preference for reward-driven stimulation under VR schedule approximating a situation of risk and excitement. This behavioral profile is consistent with a state of stable moderate hyperdopaminergia and partial desensitization of receptor systems, leading to rigidity of actions and impaired probabilistic choice.

## 4. Materials and Methods

Animal selection. The study was conducted on sexually mature rats aged 11–12 weeks at the beginning of the experiments, with body weights ranging from 250 to 350 g. Behavioral experiments were performed in wild-type Wistar (WT; *n* = 20) and heterozy-gous dopamine transporter knockout (DAT-HET; *n* = 18) rats. Homozygous dopamine transporter knockout rats (DAT-KO; *n* = 5) were used exclusively for fast-scan cyclic volt-ammetry (FSCV) experiments.

Animals were housed in groups of five in standard plastic cages (53 × 32 × 19 cm) containing wood-shaving bedding under controlled vivarium conditions, including a 12 h light/12 h dark cycle, an ambient temperature of 22 ± 2 °C, and a relative humidity of 50–55%. Behavioral testing was conducted in a separate experimental room during the dark phase of the cycle (4:00 p.m.–8:00 p.m.) while maintaining environmental conditions identical to those used for housing.

Rats with complete and partial knockout of the *DAT* gene were derived from line provided by R. R. Gainetdinov (Institute of Translational Biomedicine, St. Petersburg State University, St. Petersburg, Russia), followed by breeding and the formation of experimental groups at the vivarium of the Federal State Budgetary Scientific Institution “Institute of Experimental Medicine.”

**Figure 7:**
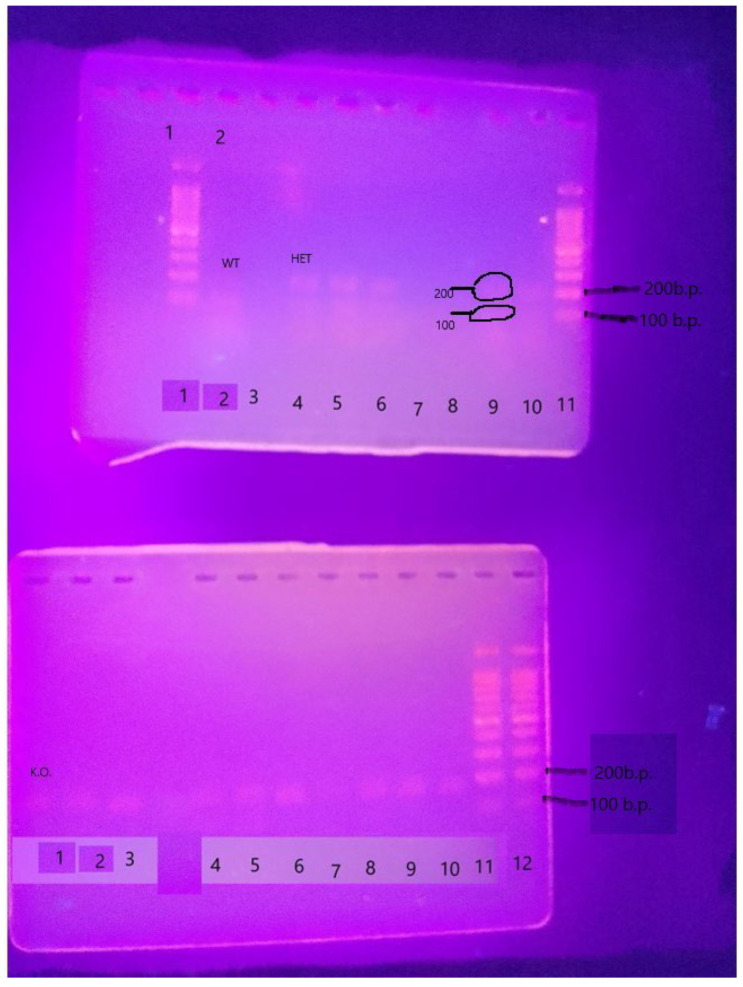
Rat DNA samples on a gel.

Molecular genetic analysis of the rats was performed at the S.V. Anichkov Department of Pharmacology of Institute of Experimental Medicine, St. Petersburg, Russia. Subsequently, PCR was performed on the DNA samples using primers targeting the *DAT* gene (200 bp fragment), followed by restriction with the BtsImutI endonuclease according to a standard protocol and agarose gel electrophoresis. When creating knockouts, a single-nucleotide deletion was introduced within the gene (in the center of the fragment obtained as a result of the reaction with primers targeting the *DAT* gene), which altered the reading frame. Consequently, the restriction site recognized by BtsImutI also disappeared. Therefore, DNA samples from wild-type rats showed a 100 bp fragment on electrophoresis, samples from knockout rats showed a 200 bp fragment, and heterozygotes for the knockout DAT-HET gene showed two fragments of 200 bp and 100 bp (Figure 7).

Note. DNA samples from (WT) rats on the gel showed a 100 bp fragment, samples from DAT-KO knockout rats showed a 200 bp fragment, and heterozygotes for the knockout gene showed two fragments of 200 bp and 100 bp. Numbers indicate gel lane numbers, letters indicate the corresponding genotype groups, and circles mark the DNA fragments used for genotype identification.

Note. The results of rat genotyping by PCR followed by restriction with the BtsImutI endonuclease are presented. WT–wild type; DAT-HET–heterozygotes; DAT-KO–homozygous knockout. Upper gel wells: 11—DNA Ladder 1000 bp–100 bp, 10—rat DAT-HET, 9—rat DAT-HET, 6—at DAT-HET, 5—rat DAT-HET, 4—rat DAT-HET, 2—rat WT, 1—DNA Ladder1000 bp−100 bp. Lower gel wells: 11,12—DNA Ladder1000 bp−100 bp, 10—rat DAT-KO, 9—rat DAT-KO, 8—rat DAT-HET, 7—rat DAT-KO, 6—rat DAT-KO, 5—rat DAT-HET, 4—rat DAT-HET, 3—rat DAT-KO, 2—rat DAT-HET, 1—rat DAT-HET.

Adapted Iowa Gambling Task. The adapted Iowa Gambling Task (IGT) for rats was administered in accordance with a protocol that represents an experimental modification of the clinical method for studying decision-making under uncertainty. The experimental setup consisted of a three-arm maze comprising a starting platform measuring 33 × 50 × 35 cm and three arms measuring 50 × 15 × 35 cm (Figure 3B). At the end of each arm was a feeder with automatic delivery of food reinforcement. Reward delivery occurred when the animal reached the feeder in the corresponding arm. After the animal returned to the starting area, the next trial became available. During testing, the number of runs to the feeder and returns to the starting chamber were recorded over a 10 min period. No additional signaling stimuli were used during the experiment.

The animals were fed daily, with access to food limited to 4 h, while water was available ad libitum. A 20 h food deprivation period was conducted before each experiment. Training in the maze was conducted daily for 21 days. Sunflower seeds were used as food reinforcement.

During the first 5 days of training, a training reinforcement ratio was used, in which each run to the feeder was followed by the receipt of a reward: when selecting arm 1, the animal received 2 seeds; arm 2, three seeds; and arm 3, four seeds. After the completion of the training phase, no experiments were conducted for the following two days. Starting on the 8th day of training, a differential reinforcement ratio was introduced, taking into account both the amount and the probability of the reward. In arm 1, reinforcement was administered under an FR-1 schedule, in which each approach to the feeder was followed by the delivery of two seeds. In arm 2, an FR-2 schedule was used: three seeds were delivered on every second approach to the feeder. In arm 3, an FR-3 schedule was used, in which four seeds were delivered on every third approach.

The animals were trained in the specified mode for two weeks. Rats that did not enter the maze arms were excluded from further analysis (no more than 15% of the total number of animals) [51].

Marble Burying Test. The tendency toward repetitive and stereotypical behaviors was assessed using this test, which is a widely used model of compulsive behavior in rodents [51]. The procedure was conducted according to a standard protocol. Each animal was placed individually in a standard cage filled with a 4–5 cm layer of sawdust. Twenty glass marbles with a diameter of 15 mm were evenly distributed on the surface of the bedding in advance. The animal was carefully placed in the cage, taking care to avoid accidentally displacing the marbles. The cage was then covered with a mesh lid, and the rat was left without food or water for 30 min, without external stimuli. At the end of the exposure, the animal was carefully removed without disturbing the arrangement of the marbles. The count was performed by two independent observers who were unaware of the animal’s group or genotype. A marble was considered “buried” if at least two-thirds of its surface was covered by bedding. For each animal, the individual number of buried marbles was calculated, after which the bedding was disposed of, and all marbles were retrieved and prepared for the next test [51].

Nestlet Shredding Test. The animal was placed in an individual cage containing a single, pre-weighed standard nestlet [52]. The cage was covered with a mesh lid; food and water were not provided during the test. The exposure lasted 30 min and took place without external stimuli. After the procedure was completed, the animal was returned to its home cage. The remaining unshredded material was carefully removed with tweezers and left to dry overnight at room temperature. The mass of the dried residue was compared to the initial mass to determine the percentage of shredded material using the formula: Percentage of shredding = (initial mass − residual mass)/initial mass × 100%. If necessary, an integrated five-point scale for assessing nest-building behavior could be used; however, in this study, the emphasis was specifically on the quantitative measure of the degree of shredding, as it primarily reflects the compulsive nature of the activity rather than the quality of the constructed nest. All remaining material and bedding were disposed of at the end of the procedure.

### 4.1. Binge-Eating Model

The eating behavior of rats was studied using a binge-eating model [11]. A chocolate-feeder mixture, consisting of 52% Nutella (Ferrero, Alba, Turin, Italy), 33% food pellets (4RF18; Mucedola; Settimo Milanese, Italy), and 5% water, was used as a treat. The treat was provided on a Monday–Wednesday–Friday schedule for 1 h, preceded by a 15 min demonstration period (during which the rats could see and smell the chocolate but not eat it). The binge-eating model required preliminary training. In the first stage (1 week), the chocolate treat was given to groups of animals (5–6 rats). In the second stage, rats were moved to individual cages, and the treat was provided until a stable level of consumption (with deviations of no more than 10%) was achieved. The second stage lasted 3 weeks. Additionally, chocolate mixture intake was normalized to each animal’s hourly chow consumption.

### 4.2. Stabilization of the Estrous Cycle

When assessing compulsive overeating in female rats, the phase of the estrous cycle and the corresponding hormonal background were considered. Stabilization was achieved through hormonal correction using a 2% (20 mg/mL) solution of synestrol (Dalhimpharm OJSC, Khabarovsk, Russia) and a 2.5% (25 mg/mL) solution of progesterone (Dalhimpharm OJSC, Khabarovsk, Russia). To synchronize the estrous cycle, female rats were administered 10 μg of estrogen benzoate 48 h and 500 μg of progesterone 2 h prior to the experiment. Cycle verification was performed 1 h before the experiment via microscopy of a vaginal smear and by measuring lumbar lordosis. The estrus phase was verified in all experimental animals following hormonal correction. The procedure for “stabilizing” the estrous cycle is described in detail in earlier publications [51].

### 4.3. Study of the Addictive Component of Behavior During Intracranial Self-Stimulation

In 5 WT rats and 5 DAT-HET rats, a stimulation electrode (a 0.2 mm thick insulated steel bipolar electrode) was implanted into the ventral tegmental area (VTA). The animals were anesthetized with zoletil-100, 50 mg/kg (Valdepharm, Val-de-Reuil, France). Electrode coordinates relative to bregma: AP = −5.3 mm, L = 0.8 mm, H = 8.2 mm [53]. Rats in the group were trained to press a pedal in a two-pedal Skinner box (St. Petersburg, Russia) (35 × 12 × 21 cm). Pedal pressing triggered electrical stimulation of the brain structure lasting 0.5 s. The number of pedal presses and the self-stimulation response threshold (μA) were analyzed over a 5 min “session.” At the beginning of training, the FR1 schedule was used, i.e., each press of either pedal was reinforced with electrical stimulation of the VTA. For stimulation, a series of rectangular pulses was applied (pulse duration 1 ms at a frequency of 100 Hz for 0.5 s). When determining threshold values in the forced mode, current was applied in increasing increments of 2 μA for 5 s each until distinct pedal pressing responses appeared. Next, the current intensity was increased by 50% of the threshold values and then decreased (in 2 μA increments with a stimulation duration of 5 s) until the subject refused to press the pedal. The procedure for determining the current intensity thresholds for self-stimulation was repeated twice. When the current values obtained in the ascending and descending modes coincided, this was considered the threshold for the self-stimulation response [53].

The rats were trained to press either of two pedals to receive a reward, with the aim of identifying a preference for one of them. One pedal was programmed to deliver a stimulus under a fixed-ratio (FR), while the other was programmed under a variable-ratio (VR). Training was conducted over 6 consecutive days with FR1 and VR1 schedules, progressively increasing the difficulty up to FR6 and VR6. When a reward was received on the FR pedal, a 60-lux light bulb lit up for 0.5 s. On the VR pedal, the light bulb flashed twice, with a total duration of 0.5 s. The number of presses and actual current stimuli for each pedal were recorded. The experiments were conducted over 3 consecutive days [54]. When using the VR ratio, the animal could not accurately determine which press would be followed by electrical stimulation of VTA. This ratio most closely approximates the excitement and risk associated with gambling [21]. Animal behavior was analyzed in separate experimental sessions. Each session lasted 5 min from the first depression on either of the two levers. Assignment of the FR and VR schedules to the left or right lever was counterbalanced across animals.

To evaluate the distribution of responses between the fixed-ratio and variable-ratio reinforcement levers, the press preference index (PPI) was calculated:

PPI = (VR_press − FR_press)/(VR_press + FR_press). (1)

This index was used as the primary behavioral outcome. Positive PPI values indicated a predominance of responses on the VR-associated lever, whereas negative values indicated a predominance of responses on the FR-associated lever. Values close to zero corresponded to an approximately even distribution of responses between the two levers.

To determine whether this difference could be explained by differences in the actual reinforcement obtained, a control additional analysis was performed using the following measure:

actΔ(VR − FR) = (VR_stim/VR_press × 100) − (FR_stim/FR_press × 100).(2)

This measure was used as a control for actual reinforcement density: it indicated how the number of stimulations per 100 responses differed between the VR and FR schedules.

### 4.4. Recording Changes in Extracellular Dopamine Concentration

The secretory activity of dopaminergic neurons in vivo was assessed using fast-scan cyclic voltammetry (FSCV), recording changes in extracellular dopamine concentration in the NAc in response to electrical stimulation of the VTA. The FSCV setup was custom-made in the engineering workshops of the Institute of Experimental Medicine (St. Petersburg, Russia). This method provides high temporal resolution (10 Hz) and spatial sensitivity determined by the dimensions of the carbon fiber microelectrode (active tip ~100 μm in length, 7 μm in diameter). Prior to implantation, each electrode was individually calibrated in vitro, during which an increase in dopamine concentration caused a dose-dependent increase in the amplitude of the voltammetric signal [54].

Animals were anesthetized with Zoletil-100 at a dose of 50 mg/kg (Valdepharm, France), and a 0.2 mm stimulation electrode was implanted in the VTA. Electrode coordinates relative to bregma: AP = −5.3 mm, L = 0.8 mm, H = 8.2 mm [55]. A carbon-fiber microelectrode in a glass capillary, used to detect dopamine release, was also implanted ipsilaterally into the NAc: AP = +2.0 mm, L = 1.2 mm, H = 6.8 mm from the skull surface. A compressed Ag/AgCl reference electrode (3 mm diameter) was placed on the skull surface (AP = +5.5 mm; L = 0) and secured with UV-curable dental acrylic. The position of the recording electrode was finely adjusted until maximum dopamine release in response to VTA stimulation was achieved, after which it was permanently fixed.

Electrical stimulation of the VTA was delivered as a series of rectangular pulses (240 μA, 1 ms duration, 100 Hz, 0.5 s duration) with a 3-min interval between series. Baseline recording continued for 60 min. FSCV parameters included a scan rate of 10 ms per cycle, a hold potential of −0.4 V, and an upper anode limit of +1.3 V, which ensured reliable separation of dopamine oxidation and reduction peaks with minimal background noise [53].

**Figure 8:**
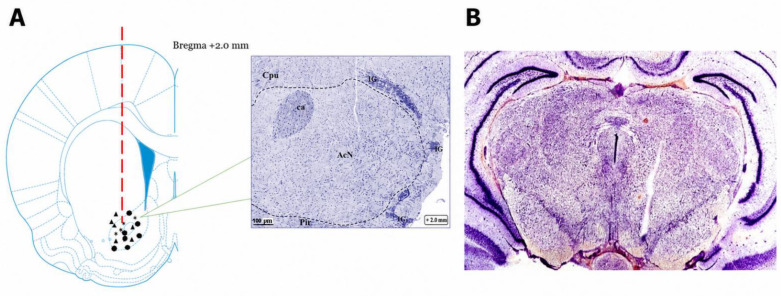
Histological localization of the nucleus accumbens and ventral tegmental area. (**A**)—Schematic coronal section and representative histological image of the nucleus accumbens region. Symbols indicate electrode locations in individual animals: circles—WT, stars—DAT-HET, triangles—DAT-KO. (**B**)—Representative histological image of the ventral tegmental area. Scale bars: 100 micrometres. Nissl staining. In panel (**A**), the blue outline indicates the schematic coronal brain section, the blue shaded area indicates the NAc region, and the red dashed line marks the brain midline. Green lines indicate the correspondence between the schematic map of recording sites and the representative histological section, while dashed black lines indicate anatomical boundaries. Letters (**A**,**B**) indicate figure panels.

Upon completion of the experiments, morphological verification of electrode placement was performed. The brain was isolated and embedded in celluloid, after which frontal sections were prepared and stained with cresyl violet using the Nissl method (Figure 8).

On the right–the anterior part of the NAc with a brain defect in the region of electrode implantation for recording at the Bregma +2.0 level. Abbreviations: NAc–nucleus accumbens, sa–anterior commissure (located within the nucleus); Cpu–striatum, IC–olfactory nuclei, Pir–piriform cortex. Stained with cresyl violet using the Nissl method. Mag. ×10, field of view ×10. Left–mapping of the investigated NAc recording sites.

Statistical analysis methods. Statistical data analysis was performed using GraphPad Prism v10.4.0 software (Boston, MA, USA). For all experimental parameters, the distribution of the samples and the homogeneity of variances were assessed beforehand. Normality of distribution was tested using the Shapiro–Wilk and Kolmogorov–Smirnov tests, and homogeneity of variances was tested using the Brown–Forsythe and Bartlett tests. In the absence of significant differences in variances, the data were analyzed using parametric statistical methods.

Given the relatively small sample size and the characteristics of the genetic models, the primary focus was on identifying stable genotype-dependent effects, as well as analyzing within-group variability. Quantitative data are presented as the mean ± standard error of the mean (SEM), with individual animal values additionally plotted on the graphs, allowing for a visual assessment of the data spread within groups.

To assess the influence of the “genotype” factor on indicators of compulsive behavior (number of marbles buried, percentage of nest material shredded) and impulsive behavior (in the Iowa Gambling Task, the total number of runs and preferences for individual arms), one-way ANOVA with the “genotype” factor (WT, DAT-HET, DAT-KO) was used. If a statistically significant main effect was detected, post hoc multiple comparisons were performed using Tukey’s test. To assess the influence of the “genotype” and “arm” factors in the Iowa Gambling Task in rats with different *DAT* genotypes (WT, DAT-HET, DAT-KO), a two-way analysis of variance (two-way ANOVA) was used, allowing assessment of both the main effects of the factors and their possible interaction.

Data are presented as the mean ± standard error of the mean (SEM). The critical significance level for all tests was *p* < 0.05. Statistically significant values in the graphs are denoted as: * *p* < 0.05, ** *p* < 0.01, *** *p* < 0.001, **** *p* < 0.0001.

#### ARRIVE Guidelines Compliance

Study Design and Experimental Groups. The study included wild-type (WT), hetero-zygous dopamine transporter knockout (DAT-HET), and homozygous dopamine trans-porter knockout (DAT-KO) rats. WT animals served as the control group. The experimental unit was a single animal.

Sample Size. Sample sizes were determined based on previous studies using dopa-mine transporter-deficient rat models and on established laboratory experience with simi-lar behavioral and neurochemical experiments. No formal a priori sample size calculation was performed.

Inclusion and Exclusion Criteria. Inclusion criteria comprised successful genotyping, normal health status, and completion of behavioral training procedures. Animals that failed to perform task requirements were excluded from the corresponding analysis. In the Iowa Gambling Task, animals that did not enter the maze arms were excluded from further analysis.

Randomisation and Blinding. Experimental groups were defined by genotype following molecular genetic verification. The order of behavioral testing and data acquisition was randomized whenever applicable. Behavioral scoring was performed by investigators blinded to genotype, and statistical analyses were conducted using coded datasets to reduce potential bias.

Outcome Measures. Primary outcome measures included indices of impulsive and compulsive behavior, binge-eating behavior, self-stimulation parameters, and extracellular dopamine release measured by fast-scan cyclic voltammetry.

All experimental procedures were approved by the Local Ethics Committee of the In-stitute of Experimental Medicine (protocol No. 4/25, date 25 December 2025) and were conducted in accordance with Directive 2010/63/EU of the European Parliament on the protection of animals used for scientific purposes.

## 5. Conclusions

The study results demonstrate that disruption of dopamine transporter function leads to a reorganization of behavioral regulation mechanisms; however, the phenotypes of partial and complete knockout differ significantly in their neurophysiological basis. In DAT-HET animals, a stable state of moderately elevated dopaminergic tone develops, in which the availability of information about the probable outcome of an action is limited, and motivationally significant stimuli begin to dominate over the assessment of outcomes. These features underscore the importance of DAT-HET in studying the neurobiological basis of impulsivity, compulsivity, and the factors underlying a predisposition to addictive behavior.

## Data Availability

The original contributions presented in this study are included in the article. Further inquiries can be directed to the corresponding author.
